# Elevation determines the productivity of large cardamom (*Amomum subulatum* Roxb.) cultivars in Sikkim Himalaya

**DOI:** 10.1038/s41598-023-47847-6

**Published:** 2023-12-07

**Authors:** Patrush Lepcha, Kailash S. Gaira, Aseesh Pandey, Santosh Kumar Chettri, Jarina Lepcha, Jhony Lepcha, Rajesh Joshi, Nakul Chettri

**Affiliations:** 1 GB Pant National Institute of Himalayan Environment, Sikkim Regional Centre, Pangthang, Post Box 24, Gangtok, 737101 Sikkim India; 2 Department of Botany, Sikkim University, P. O, Tadong, Gangtok, Sikkim-737102 India; 3 GB Pant National Institute of Himalayan Environment, Kosi-Katarmal, Almora, 263643 Uttarakhand India; 4 International Centre for Integrated Mountain Development, Post Box. 3226, Kathmandu, Nepal

**Keywords:** Plant sciences, Environmental sciences

## Abstract

Large cardamom (*Amomum subulatum* Roxb.) is an economically important cash crop that provides a livelihood option for the rural communities in Sikkim Himalaya. However, its production has declined drastically over the past few decades due to climate change and other factors affecting the livelihood of marginal cardamom-dependent farmers in the region. Climate change causes a shift in elevational distributions of mountain species, and it is pivotal to understand its effect on yield and yield-related traits for economically important plant species like large cardamom. For this, we randomly studied 41 large cardamom cultivation sites in Sikkim (India) with elevations ranging between 975 and 2069 m asl and evaluated the yield-related traits (number of capsules per spike, capsule length, capsule width, fresh capsule weight, dry capsule weight, number of seeds per locule, fresh seed weight, and dry seed weight) in five cultivars (Dzongu Golsey, Sawney, Seremna, Ramsey, and Varlangey). We observed a significant variability (*P* < 0.05) for morphometric traits in each of the five cultivars cultivated in different elevations. The cultivation of low-elevation cultivars like Seremna and Dzongu Golsey (suitable in elevation < 975 m) has shifted upward to mid (975–1515 m) and high-elevation (> 1515 m), while cultivation of high-elevation Ramsey cultivar (suitable in elevation > 1515 m) has shifted downward (< 1515 m). The Dzongu Golsey, Sawney, and Seremna cultivated in mid-elevation (975–1515 m) performed better in terms of yield-related traits than the same cultivars cultivated in high-elevation (> 1515 m) and showed moderate to high negative correlation between elevation and yield-related traits, indicating the negative effect of elevation on their yield. Likewise, Ramsey and Varlangey cultivated in high elevation (> 1515 m) performed better than the one cultivated in mid-elevation (975–1515 m) and depicted moderate to high positive correlation between elevation and yield-related traits, suggesting a positive influence of elevation on their yield. Although there is an elevational shift in the cultivation of large cardamom cultivars, the elevation influences the performance of the large cardamom cultivars, and it also suggests cultivating the cultivars in their suitable elevation range for better productivity.

## Introduction

The Large cardamom (*Amomum subulatum* Roxb., family-Zingiberaceae), is one of the most ancient spices^1^ and the third most expensive spice after saffron and vanilla^2^. The large cardamom is a major source of income for farmers in the eastern Himalayan region, including Sikkim and West Bengal in India; eastern Nepal; and southern Bhutan^3,4^. It is native to the eastern Himalayan region and domesticated first by the indigenous Lepcha tribe of Sikkim in India, and later was passed on to the neighboring district of Darjeeling in West Bengal (India), southern Bhutan, and eastern Nepal^3^. The crop plant is a perennial shrub with several tillers consisting of pseudo-stems with leaves appearing on the upper part and an inflorescence (spike) arising from the rhizome. It is typically a cross-pollinated plant but is also capable of self-pollination^3^. It is a shade-loving (sciophyte) species traditionally cultivated in a mixed agroforestry system, preferably with the Himalayan alder plant (*Alnus nepalensis* D. Don)^3,5,6^. The seeds of the large cardamom are used to add flavor to various food and vegetable recipes and contain 3% essential oil, which is rich in cineole as well as possesses medicinal properties like a cardiac stimulant, diuretic, carminative, stomachic, and anthelmintic^1^. Because of being a high-value cash crop, it is often termed a “currency crop”^7^.

There are about 18 cultivars of large cardamom available in India, Nepal, and Bhutan, namely—Dzongu Golsey, Sawney, Seremna, Ramsey, Ramla, Varlangey, Chivey, Gardo, Ramnag, Madhusey, Seto Golsey, Slant Golsey, Red Sawney, Green Sawney, Dambersai, Jhirmale, Kantidas, and Mingney and two high yielding varieties- ICRI Sikkim 1 and ICRI Sikkim 2, developed by Indian Cardamom Research Institute (ICRI), Regional Station, Spices Board Tadong (Sikkim, India)^5,8–11^. Out of these, six cultivars, such as Dzongu Golsey, Sawney, Seremna, Ramsey, Ramla, and Varlangey are the popular choice for the farmers^10^. High phenotypic and genetic diversity were reported in these cultivars of large cardamom^12–14^. Such diverse genetic resource is a prerequisite for any crop improvement program. However, the prevalence of high diversity in the large cardamom cultivars needs to be properly utilized to develop improved cultivars through conventional and modern molecular breeding techniques.

At present, India, Nepal, and Bhutan are major contributors to the world's production of large cardamom^15–17^. In India, Sikkim is the largest producer of large cardamom contributing about 80% of the total production in the country^8,18,19^. Large cardamom was the primary driver of the agricultural economy in Sikkim Himalaya before 1997 where this crop contributed half of the household income, but this contribution declined to 29% during 2016 with low productivity in more than half of the cultivated area^20^. Over the past decade, the cultivated area and productivity of the large cardamom have declined drastically due to climate change, increased diseases and pests and anthropogenic pressure^8,19–23^. This has affected the income of marginal and cardamom-dependent farmers in the eastern Himalayan region and jeopardized their livelihoods^15,24–27^. Despite this loss, there is a vast knowledge gap about the impact of climate change on large cardamom cultivation^28^ and the impact of elevational shifts on large cardamom production caused by climate change.

Each of the cultivars of large cardamom is suitable for cultivation at a specific elevational range and is adapted to local environmental extremes like water deficit and frost^8^. For example, Seremna cultivar is cultivated in low elevation (< 975 m), Dzongu Golsey is suitable from low (< 975 m) to 1300 m elevation, Sawney and Varlangey are suitable in both the mid (975–1515 m) and the high (> 1515 m) elevation, and Ramsey is best suited at high elevations (above 1515 m), etc^5,8,10,11^. But the agricultural ecosystems and crop suitability have changed remarkably across the globe due to climate change^29^. In the Nordic region (Northern Europe), the grass and the cereal distribution is expected to shift by up to 92.8 and 178.7 km, depending on the extent of the climate change scenario^29^ and some crop species are being introduced to new areas^30^. An upward shift of 53 plant species at Mt. Gongga was observed in response to climate change^31^, and several other studies have also generated a similar trend of an upward shift of the plant species in response to climate change^32–37^.

Sikkim (India) lies in the mountainous region (Eastern Himalaya), and this region of the world is most vulnerable to climate change^38,39^. Therefore, the region is prone to the consequences of climate change, such as a shift in elevational distributions of mountain plant species like large cardamom. However, whether climate change is causing the elevational shift in cultivating large cardamom cultivars has not been studied. Elevation affects the growth and development of plants^40^, and it can significantly affect temperature, humidity, sunlight hours, UV-B radiation, water deficits, and other environmental factors^41^. Such environmental factors exposed to plants and water and nutrients absorbed by them differ at different elevations^42,43^. As a consequence, plants alter their physiological and morphological characteristics in response to environmental conditions regulated by the elevational gradients^44,45^. The locally adapted plants require an optimum elevation for biomass production and the net photosynthetic rate and enzymatic activity decrease or increase when the optimum elevation is changed^46–48^. Thus, if there is an elevational shift in the cultivation of large cardamom, it is essential to analyze large cardamom performance across the elevations because the income of several marginal farmers in the Eastern Himalayan region depends on this cash crop.

Considering the above, an attempt was performed to study the twelve morphological traits, including eight yield-related traits, in each of the five cultivars grown in 41 locations in Sikkim at varying elevations ranging from 975 to 2069 m (asl). The objectives of the study were to determine (1) the shift in the cultivation of each large cultivar to different elevations, (2) the morphometric variability in each large cardamom cultivar cultivated at different elevations, and (3) the performance of five large cardamom cultivars (Dzongu Golsey, Sawney, Seremna, Ramsey, and Varlangey) in two different elevational ranges, i.e. mid-elevation (975–1515 m) and high elevation (> 1515 m).

## Results

### Morphometric variability among large cardamom cultivars

Out of six popular and widely cultivated large cardamom cultivars, five cultivars: Varlangey, Ramsey, Seremna, Sawney, and Dzongu Golsey (excluding Ramla), were recorded from 41 large sites with an elevation ranging between 975 and 2069 m, spread over three districts (South, East, and North) in Sikkim, India (Table S1). Out of 41 sites, Dzongu Golsey was found at four sites, Sawney at five sites, Seremna at six sites, Ramsey at ten sites, and Varlangey at sixteen sites (Table S1). The data for twelve morphometric traits such as plant height, number of leaves per tiller, leaf length, leaf width, number of capsules per spike, capsule length, capsule width, fresh capsule weight, dry capsule weight, number of seeds per locule, fresh seed weight, and dry seed weight were recorded from above five large cardamom cultivars grown at different sites (Table S2). The method of measurement of each trait is provided in detail in the methods section. Out of twelve morphometric traits evaluated in the study, eight were yield-related traits (number of capsules per spike, capsule length, capsule width, fresh capsule weight, dry capsule weight, number of seeds per locule, fresh seed weight, and dry seed weight).

To analyze the phenotypic diversity existing among five cultivars of the large cardamom, we analyzed the variability in each of the twelve morphometric traits recorded at all 41 sites. Except for leaf length the one-way analysis of variance (ANOVA) revealed significant variability (*P* < 0.05) for traits such as plant height (1.98 m), number of leaves per tiller (9.00), leaf length (60.61 cm), (leaf width 8.64 cm), number of capsules per spike (12.00), capsule length (23.44 mm), capsule width (16.92 mm), fresh capsule weight (46.04 g), dry capsule weight (6.72 g), number of seeds per locule (14.00), fresh seed weight (28.96 g), and dry seed weight (9.85 g) among the cultivars (Table 1). Out of the total traits analyzed in this study, dry weight of seed, which ranged from 1.36 to 32.16 g, had the maximum coefficient of variation (CV = 43.66%), and the capsule width, which ranged from 12.52 to 21.96 g had the minimum coefficient of variation (CV = 12.83%).

**Table 1:** Variability for morphometric traits among the five large cardamom cultivars evaluated from 41 study sites in Sikkim.

Traits (unit)	Abv	Min	Max	Mean	CV%	Sig
Plant height (m)	PH	0.46	3.50	1.98 ± 0.046	25.85	***
No. of leaves per tiller	NLpT	5.00	13.00	9.00 ± 0.142	17.28	***
Leaf length (cm)	LL	38.30	85.50	60.61 ± 0.749	13.65	ns
Leaf width (cm)	LW	6.11	12.17	8.64 ± 0.102	12.99	**
No. capsule per spike	NCpS	4.00	21.00	12.00 ± 0.294	27.21	**
Capsule length (mm)	CL	16.59	33.57	23.44 ± 0.302	14.25	**
Capsule width (mm)	CW	12.52	21.96	16.92 ± 0.196	12.83	*
Fresh weight of capsule (g)	FWC	18.94	88.48	46.04 ± 1.419	34.05	***
Dry weight of capsule (g)	DWC	1.56	12.90	6.72 ± 0.231	37.95	**
No. of seed per locule	NSpL	6.00	31.00	14.00 ± 0.472	36.08	**
Fresh seed weight (g)	FSW	10.38	79.44	28.96 ± 0.828	31.58	**
Dry seed weight (g)	DSW	1.36	32.16	9.85 ± 0.390	43.66	*

Abv, abbreviations of traits; Min, minimum; Max, maximum; CV, coefficient of variation; Sig, significance based on one-way ANOVA, ± standard error, *significant at P < 0.05, **significant at P < 0.01, ***significant at P < 0.001, ^ns^non-significant.

On comparing the average values of all twelve morphometric traits recorded from each of the five cultivars of large cardamom in the overall study area (Table 2), the Varlangey cultivar was found to be the tallest among the large cardamom cultivars with an average plant height of 2.19 m bearing a maximum number of leaves (an average of 10). In contrast, the Dzongu Golsey was the shortest, with an average height of 1.45 m, bearing a minimum number of leaves (approximately 7). The highest number of capsules per spike (13.0) was observed in the Varlangey cultivar, followed by Sawney, Dzongu Golsey (12 each), Seremna and Ramsey (11 each). The highest value for capsule length (24.98 mm) was also recorded in Varlangey, followed by Sawney (23.37 mm), and similar values in Seremna (22.57 mm), Ramsey (22.37 m) and Dzongu Golsey (21.35 mm). Capsule width was similar in Varlangey (17.57 mm), Seremna (17.18 mm), Dzongu Golsey (17.10 mm), and slightly less in Sawney (16.17 mm) and Ramsey (15.99). Similar fresh capsule weight was observed in Sawney (38.76 g), Seremna (38.54 g), Ramsey (38.50 g), and Dzongu Golsey, but the highest was in Varlangey (57.59 g). Fresh seed weight was highest in Varlangey (33.28 g) but similar in Seremna and Sawney (26.92 g and 26.81 g, respectively) and Ramsey and Dzongu Golsey (25.86 g and 25.08 g, respectively). The value for dry weight of the capsule was highest in Varlangey (7.67 g), followed by Seremna (7.31 g), Dzongu Golsey (6.40 g), Ramsey (5.68 g), and Sawney (5.17 g). Likewise, the maximum value for dry seed weight was observed in Seremna (11.40 g), followed by Varlangey (10.23 g), Dzongu Golsey (9.67 g), Ramsey (9.59 g), and Sawney (7.28 g). The cultivar Varlangey had the maximum capsules per spike (average 13), and Ramsey had the minimum (average 11). Results revealed that the Varlangey cultivar included the highest values for most yield-related traits compared to the other cultivars.

**Table 2:** Average values of 12 morphometric traits in each of the five cultivars of large cardamom evaluated from 41 study sites in Sikkim.

Traits	Dzongu Golsey	Sawney	Seremna	Ramsey	Varlangey
	Mean ± SE	Mean ± SE	Mean ± SE	Mean ± SE	Mean ± SE
PH (m)	1.45 ± 0.18	2.01 ± 0.05	1.81 ± 0.12	1.96 ± 0.06	2.19 ± 0.08
NLpT	7.00 ± 0.51	9.00 ± 0.30	8.00 ± 0.22	8.00 ± 0.25	10.00 ± 0.21
LL (cm)	57.14 ± 2.99	62.12 ± 1.68	62.08 ± 1.82	59.07 ± 1.10	61.44 ± 1.36
LW (cm)	8.46 ± 0.41	8.02 ± 0.26	9.34 ± 0.31	8.31 ± 0.15	8.81 ± 0.15
NCpS	12.00 ± 0.60	12.00 ± 1.03	11.00 ± 0.73	11.00 ± 0.50	13.00 ± 0.52
CL (mm)	21.35 ± 0.68	23.37 ± 0.84	22.57 ± 0.82	22.37 ± 0.50	24.98 ± 0.48
CW (mm)	17.10 ± 0.71	16.17 ± 0.44	17.18 ± 0.55	15.99 ± 0.30	17.57 ± 0.32
FCW (g)	38.46 ± 3.02	38.76 ± 1.61	38.54 ± 1.96	38.50 ± 1.66	57.59 ± 2.49
DWC (g)	6.40 ± 0.79	5.17 ± 0.70	7.31 ± 0.40	5.68 ± 0.48	7.67 ± 0.33
NSpL	16.00 ± 2.29	16.00 ± 1.23	15.00 ± 1.41	11.00 ± 0.73	15.00 ± 0.63
FSW (g)	25.08 ± 1.79	26.81 ± 1.82	26.92 ± 1.57	25.86 ± 1.18	33.28 ± 1.58
DSW (g)	9.67 ± 1.23	7.28 ± 1.00	11.40 ± 0.80	9.59 ± 0.73	10.23 ± 0.68

Note: Abbreviation of traits are same as in Table 1, SE = standard error.

### Morphometric variability among large cardamom grown at different elevations

**Figure 1:**
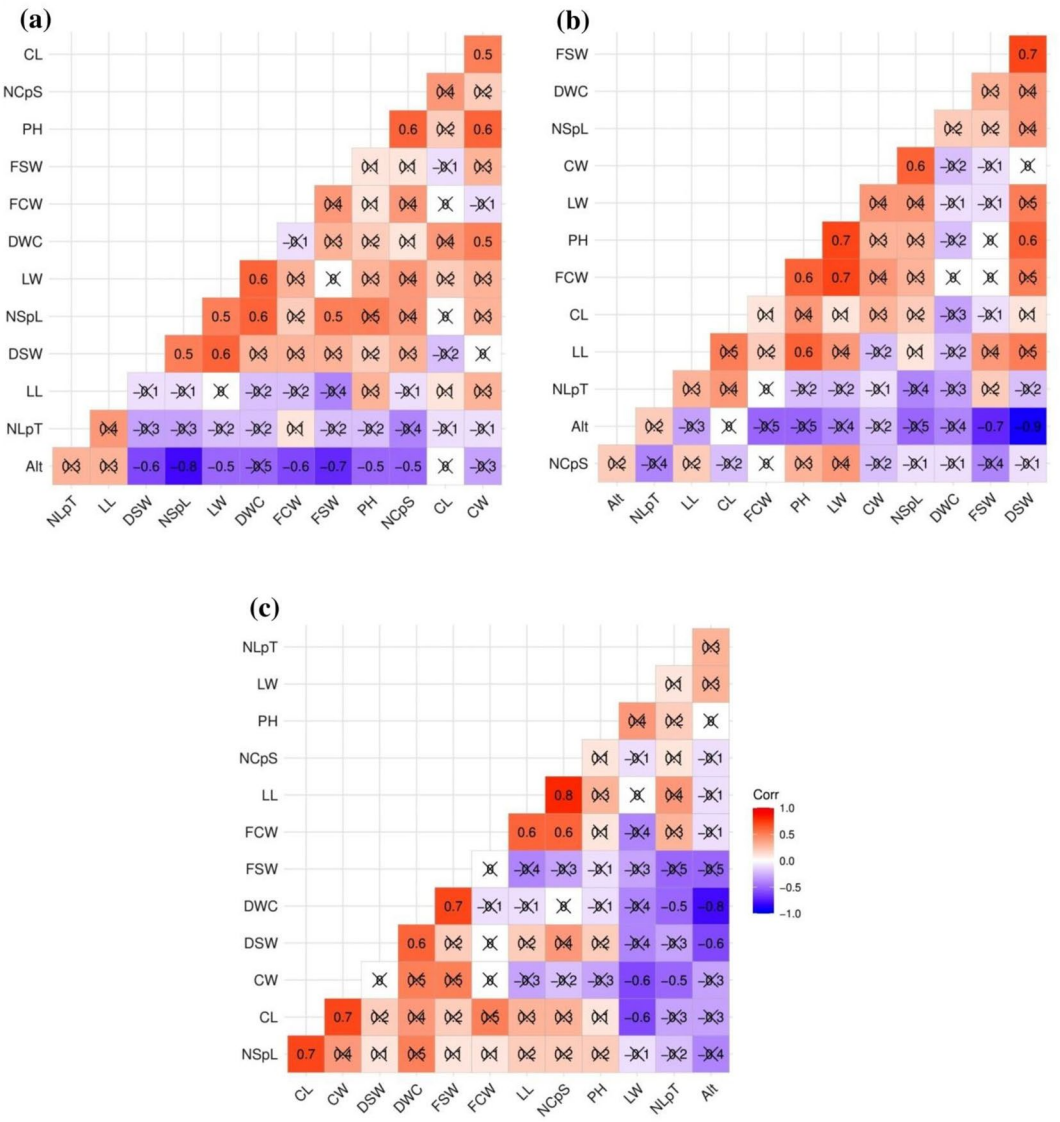
Correlation between 12 morphometric traits and elevation in low- to mid-elevation large cardamom cultivars: (**a**) Seremna, (**b**) Dzongu Golsey, (**c**) Sawney Note: Alt, altitude/elevation; PH, plant height; NLpT, number of leaves per tiller; LL, leaf length; LW, leaf width; NCpS, number of capsules per spike; CL, capsule length; CW, capsule width; FCW, fresh capsule weight; DCW, dry capsule weight; NSpL, number of seeds per locule; FSW, fresh seed weight; DWC, dry seed weight.

The elevations of our study sites ranged between 975 and 2069 m. Therefore, we performed one-way ANOVA to determine the morphometric variability within the five cultivars grown at different elevations (Table 3). Seremna cultivar was found at the six plantation sites (three each in the South and East Districts) with an elevation ranging between 975 and 2069 m (Tables S1 and S2). The morphometric traits such as plant height, leaf length, leaf width, number of capsules per spike, number of seeds per locule, fresh seed weight, and dry seed weight differed significantly (*P* < 0.05) within Seremna cultivated at different elevations (Table 3). However, no significant difference was observed for traits such as number of leaves per tiller, capsule length, capsule width, fresh capsule weight, and dry capsule weight (*P* > 0.05). We also performed a correlation analysis coupled with two-tailed t-test to determine the relationship between the morphometric traits of each cultivar of large cardamom and the elevation (Fig. 1). In the case of Seremna, the elevation had a significantly high negative correlation (*r* > − 0.70; *P* < 0.05) with the number of seeds per locule (*r* = − 0.80) and fresh seed weight (− 0.70), and a moderate negative correlation (− 0.30 < *r* < − 0.70; *P* < 0.05) with fresh capsule weight (-0.60), dry seed weight (− 0.60), plant height (− 0.50), leaf width (− 0.50), number of capsules per spike (− 0.50), respectively (Fig. 1a).

**Table 3:** One way-ANOVA based morphometric variability for twelve traits within each of the five cultivars grown at different elevations.

Traits	Dzongu Golsey		Sawney		Seremna		Ramsey		Varlangey	
	*P*-value	Sig	*P*-value	Sig	*P*-value	Sig	*P*-value	Sig	*P*-value	Sig
PH	0.011	*	0.982	ns	0.000	***	0.833	ns	0.027	*
NLpT	0.023	*	0.154	ns	0.234	ns	0.939	ns	0.001	**
LL	0.120	ns	0.404	ns	0.011	*	0.225	ns	0.018	*
LW	0.015	*	0.672	ns	0.001	**	0.610	ns	0.107	ns
NCpS	0.144	ns	0.347	ns	0.022	*	0.107	ns	0.017	*
CL	0.346	ns	0.461	ns	0.127	ns	0.212	ns	0.055	ns
CW	0.271	ns	0.233	ns	0.133	ns	0.825	ns	0.064	ns
FCW	0.212	ns	0.266	ns	0.217	ns	0.716	ns	0.002	**
DWC	0.581	ns	0.001	**	0.162	ns	0.009	**	0.067	ns
NSpL	0.100	ns	0.082	ns	0.002	**	0.002	**	0.055	ns
FSW	0.007	**	0.053	ns	0.000	***	0.457	ns	0.000	***
DSW	0.001	**	0.161	ns	0.015	*	0.022	*	0.000	***

Note: Abbreviation of traits are same as in Table 1, Sig = significance, ^ns^non-significant, *significant at the 0.05 level, **significant at the 0.01 level, ***significant at the 0.001 level.

Similarly, the Dzongu Golsey cultivar was found in four plantation sites (two each in South and East Districts) between elevational range of 1100–1842 m (Table S1 and S2). Significant variability (*P* < 0.05) was observed for plant height, number of leaves per tiller, leaf width, fresh seed weight, and dry seed weight within Dzongu Golsey grown at four different elevations (Table 3). While there was no significant difference observed for remaining traits (*P* > 0.05). The correlation analysis revealed a significantly high negative correlation (*P* < 0.05) between elevation and dry seed weight (− 0.90) and fresh seed weight (− 0.70), respectively but correlation with remaining other traits was not significant (*P* > 0.05) (Fig. 1b). Sawney cultivar was recorded from five plantation sites (3 from East and 2 from South District) located between the elevation ranges 1441–1926 m (Table S1). A significant difference (*P* < 0.05) in morphometric traits within Swaney cultivars from five elevations was recorded only for the dry capsule weight (Table 3) and significant negative correlation (*P* < 0.05) was observed between elevation and dry capsule weight (− 0.80) and dry seed weight (− 0.60), respectively (Fig. 1c). Therefore, these significantly high to moderate negative correlations between elevation and different yield-attributing traits in Seremna, Dzongu Golsey, and Sawney cultivars potentially suggest that the increasing elevation has negative influence on the yield-attributing traits in these cultivars.

**Figure 2:**
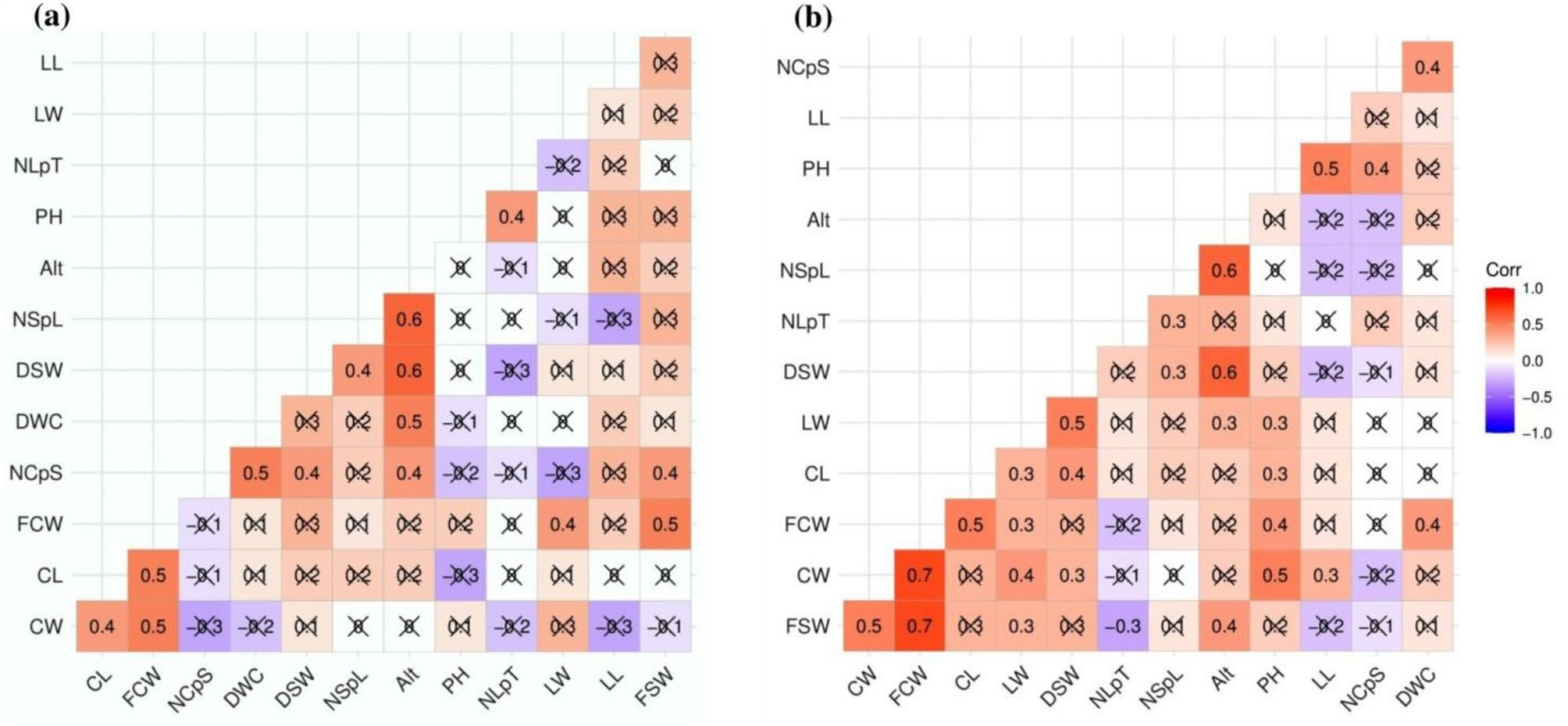
Correlation between twelve morphometric traits and elevation in mid- to high-elevation large cardamom cultivars: (**a**) Ramsey, (**b**) Varlangey. Note: Alt, altitude/elevation; PH, plant height; NLpT, number of leaves per tiller; LL, leaf length; LW, leaf width; NCpS, number of capsules per spike; CL, capsule length; CW, capsule width; FCW, fresh capsule weight; DCW, dry capsule weight; NSpL, number of seeds per locule; FSW, fresh seed weight; DWC, dry seed weight.

The Ramsey cultivar was found at ten plantation sites (six in east and four in north Sikkim) with elevations ranging from 1209 to 1783 m (Tables S1 and S2). We observed significant differences (*P* < 0.05) in plant height, capsule width, and fresh seed weight within Ramey cultivated at different elevations and for the rest of the traits. While for the rest of the trait differences were not significant (Table 3). There was a significant moderate positive correlation (*P* < 0.05) between elevation and the number of capsules per spike (0.40), number of seeds per locule (0.60), dry capsule weight (0.50), and dry seed weight (0.60), respectively (Fig. 2a). The Varlangey cultivar was found at sixteen sites representing wide elevational ranges from 1495 to 2069 m (Tables S1 and S2). Significant differences (*P* < 0.05) were observed in plant height, number of leaves per tiller, leaf length, number of capsules per spike, fresh capsule weight, fresh seed weight, and dry seed weight (Table 3). There was a significant moderate positive correlation (*P* < 0.05) between elevation and leaf width (0.30), number of seeds per locule (0.60), fresh seed weight (0.40), and dry seed weight (0.60), respectively (Fig. 2b). These significantly moderate correlations between elevation and different yield-attributing traits in Ramsey and Varlangey potentially suggest that the increasing elevation has positive influence on yield-attributing traits for these two cultivars.

### Variability in yield-related traits at two elevational ranges

**Figure 3:**
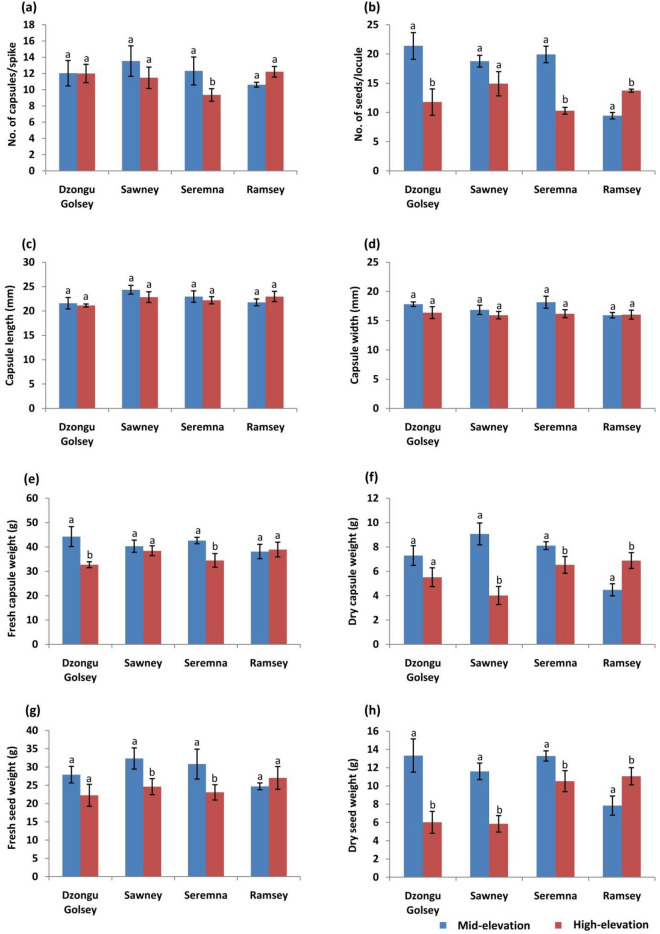
Comparison of yield-related traits within large cardamom cultivars cultivated at two elevational ranges i.e., mid-elevation (975–1500 m) and high elevation (above 1500 m): (**a**) number of capsules per spike, (**b**) number of seeds per locule, (**c**) capsule length, (**d**) capsule width, (**e**) fresh capsule weight, (**f**) dry capsule weight, (**g**) Fresh seed weight, (**h**) dry seed weight. Note: the same alphabet on top of the graph means no significant difference and a different alphabet means a significant difference in trait.

To determine the performance of large cardamom cultivars at different elevations, we divided the cardamom plantation sites of each cultivar into two elevational ranges: mid-elevation (975–1515 m) and high elevation (above 1515 m) according to the Large Cardamom Guide—2015^10^ and we compared the yield-related traits for each cultivar grown at these two elevational ranges (Fig. 3) (Table S3). Therefore, from here onwards, study sites with an elevation between 975 and 1515 m will be referred to as mid-elevation, and elevation > 1515 m will be denoted as high elevation.

The Seremna cultivars are best suited at low elevations (below 975 m)^5,8,10,11^. However, this study showed that they are being grown at sites with an elevation ranging between 975 and 2069 m (Tables S1 and S2), which falls between mid to high elevations based on the above mentioned distinction representing an upward elevational shift in the cultivation of the Seremna cultivar, i.e., from low (< 975 m) to mid (975–1515 m) and high elevations (> 1515 m). On comparing the yield-related traits for the Seremna cultivar grown at mid and high elevations, we observed that the average values of yield-related traits like the number of capsules per spike (12), fresh capsule weight (42.62 g), dry capsule weight (8.10 g), number of seeds per locule (20), fresh seed weight (30.79 g), and dry seed weight (13.28) of Seremna grown within mid-elevation (975–1515 m) were significantly higher (*P* < 0.05) than those grown at high elevation i.e., above 1500 m (number of capsules per spike = 9, fresh capsule weight = 34.46 g, dry capsule weight 6.52 g, number of seeds per locule = 10), fresh seed weight = 23.05 g, and dry seed weight = 10.52 g) (Fig. 3) (Table S3). The average capsule width was slightly high in the Seremna grown at mid-elevation (18.16 mm) compared to high elevation (16.20 mm), however, the difference was not significant (*P* > 0.05) (Fig. 3) (Table S3). The average capsule length was similar in both the elevations (approximately 23 mm each).

Similarly, the Dzongu Golsey cultivar is suitable below 975 m^5,8,10,11^ and according to Shrestha and Shrestha^5^, it is also suitable below 1300 m. In our study, the elevation of Dzongu Golsey cultivation sites ranged from 1100 to 1842 m (Tables S1 and S2), in which two sites are located above 1515 m, i.e., high elevation. Thus, this cultivar also showed upward elevational shift in the cultivation. The average number of seeds per locule (21), fresh capsule weight (44.24 g), and dry seed weight (13.33 g) were significantly greater (*P* < 0.05) of Dzongu Golsey grown at mid-elevation was significantly higher than those grown at high elevation (number of seeds per locule = 12, fresh capsule weight = 32.68 g, and dry seed weight = 6.01 g) (Fig. 3) (Table S3). The average capsule width (17.81 g), dry capsule weight (7.29 g), and fresh capsule weight (44.24 g) were slightly high in Dzongu Golsey of mid-elevation compared to high elevation (capsule width = 16.38, dry capsule weight = 5.51 g, and fresh capsule weight = 32.68 g), however, differences were not significant (*P* > 0.05). The average number of capsules per spike and capsule length was similar in both elevations (12 and 21 mm, respectively).

Sawney cultivar, which is suitable in both the mid (975–1515 m) and the high (> 1515 m) elevation^5,8,10,11^, was found in the sites with an elevation between 1441 and 1926 m (Tables S1 and S2). In this cultivar, the average values for the number of seeds per locule (19), fresh seed weight (32.33 g), and dry seed weight (11.59) were significantly higher (*P* < 0.05) for the mid-elevation sites than that grown at high elevation (number of seeds per locule = 15, fresh seed weight 24.62 g, and dry seed weight 5.85 g) (Fig. 3) (Table S3). Although we observe a slightly higher average number of capsules per spike (14), capsule length (24.35 mm), capsule width (16.85 mm), fresh capsule weight (40.29 g), and dry capsule weight (9.07) in Sawney cultivated at mid-elevation than that of high elevation (number of capsule per spike = 11, capsule length = 22.83 mm, capsule width = 15.94 mm, fresh capsule weight = 38.41 g, and dry capsule weight = 4.01 g), differences were not significantly (*P* > 0.05).

The Ramsey cultivar is best suited at high elevations (above 1515 m)^5,8,10,11^, but this study showed that its cultivation has shifted to sites with an elevation of less than 1515 m (between 1209 and 1783 m) (Table S1 and S2). This cultivar showed a downward elevational shift in the cultivation i.e., from high (> 1515 m) to mid-elevations (975–1515 m). On comparing the yield-related traits grown at mid and high elevation, the Ramsey cultivar grown at high elevation showed significantly higher (*P* < 0.05) values for dry capsule weight (6.88 g), number of seeds per locule (14), and dry seed weight (11.06 g) than that of mid-elevation (dry capsule weight = 4.48 g, number of seeds per locule = 9, and dry seed weight = 7.84 g) (Fig. 3) (Table S3). Although yield-related traits like the number of capsules per spike (12), capsule length (22.97 mm), capsule width (16.04 mm), and fresh seed weight (27.02 g) were slightly higher in the Ramsey grown at high elevation than that of mid-elevation (number of capsules per spike = 11, capsule length = 21.77 mm, capsule width = 15.94 mm, and fresh seed weight = 24.70 g), the differences were non-significant (Fig. 3) (Table S3). Values for fresh capsule weight were similar in both elevations i.e. approximately 38 g each. In this study, almost all the Varlangey cultivar were recorded from a high elevation i.e., > 1515 m, which is its best-suited elevation (Tables S1 and S2). Thus, compared to the other four cultivars, Varlangey performed well in terms of height (tallest among the large cardamom cultivars), bearing a maximum number of leaves (an average of 10), maximum average values for yield-related traits like the number of capsules per spike (13), capsule length (24.98 mm), capsule width (17.57 mm), fresh capsule weight (57.59 g), dry capsule weight (7.67 g), fresh seed weight (33.28) and second highest value for dry seed weight (10.23 g) (Table 2).

## Discussion

Large cardamom (*Amomum subulatum* Roxb.) is an economically important cash crop that provides a livelihood option for the rural communities in the Eastern Himalayan region, including Sikkim and West Bengal in India; eastern Nepal; and Southern Bhutan^3,4^. Out of 18 large cardamom cultivars available in India (Sikkim, Darjeeling), Nepal, and Bhutan^5,8,10,11^, Dzongu Golsey, Sawney, Seremna, Ramsey, Ramla, and Varlangey are the most popular among farmers and more widely cultivated than the other remaining cultivars^10^. In this study, we recorded five popular large cardamom cultivars (Dzongu Golsey, Sawney, Seremna, Ramsey, and Varlangey) from 41 sites with an elevation ranging between 975 and 2096 m. We observed significant phenotypic variability (*P* < 0.05) for traits such as plant height, number of leaves per tiller, leaf length, leaf width, number of capsules per spike, capsule length, capsule width, fresh capsule weight, dry capsule weight, number of seeds per locule, fresh seed weight, and dry seed weight among these five cultivars. Similar results of high phenotypic and genetic diversity were reported among different cultivars of large cardamom in Sikkim and Nepal^12–14^. The phenotypic and genetic diversity in crop plants is a prerequisite for any crop improvement program because it allows breeders to produce novel and improved cultivars with desirable traits^49^. However, such a high genetic diversity prevalent in the large cardamom cultivars has yet to be appropriately exploited to develop improved cultivars using conventional and molecular breeding methods. A few efforts from the local communities of Sikkim led to the development of Seremna and Dzongu Golsey cultivars, which are high-yielding, location-specific, and disease and pests resistant, developed by the Limboo tribes of Hee-Bermiok, West Sikkim and Lepcha tribe of Dzongu, North Sikkim, respectively^8,9^. The Indian Cardamom Research Institute, Regional Station, Spices Board Tadong (Sikkim) also released two high-yielding varieties (ICRI Sikkim 1 and ICRI Sikkim 2) through selection from the cultivar Sawney in the year 2004^8–10^. More such breeding effort is needed from the scientific community to develop large cardamom cultivars resilient to biotic and abiotic stresses brought about by climate change, which would potentially improve the productivity of large cardamom and increase the declining income of marginal cardamom dependant farmers of the mountainous regions of Sikkim, Darjeeling (India), Nepal, and Bhutan.

Each of the cultivars of large cardamom is suitable for cultivation at a specific elevational range and is adapted to local environmental extremes like water deficit and frost^8^. According to the previous reports, Sawney cultivar is suitable in both the mid (975–1515 m) and the high (> 1515 m) elevation^5,8,10,11^. The current study also supports its preferred elevational range i.e., within mid to high (1441 m 1926 m). Varlangey cultivar can be grown both at mid and high elevations but performs well at high elevations^5,8,10,11^. Our results confirm that Varlangey grows well at high elevation (i.e. > 1515 m). Unlike Sawney and Varlangey, we observed a change in the elevational range for Seremna, Dzongu Golsey, and Ramasey cultivars. For example, the Seremna cultivar was previously cultivated in low elevation, i.e., below 975 m^5,8,10,11^ and in this study, it was found in sites with elevations ranging between 975 and 2069 m. Similarly, the Dzongu Golsey cultivar is suitable from low to 1300 m^5,8,10,11^ and was recorded from the site with an elevation ranging between 1100 and 1842 m. Such results indicates that the cultivation of these two low-elevation cultivars (Seremna and Dzongu Golsey) has gradually shifted to new areas with higher elevations. Likewise, the Ramsey cultivar was best suited at high elevations (above 1515 m)^5,8,10,11^, but in this study, it was found at the elevation between 1209 and 1783 m, i.e., downward shift in the Ramsey cultivation. The large cardamom is a high-value, low-volume, non-perishable perennial cash crop^53^. However, the large cardamom cultivation has declined drastically over the past decade due to several factors like diseases and pests and erratic climatic conditions^8,19–23^. To achieve a high yield, farmers are trying different methods like cultivating multiple cultivars of large cardamom on the same field, shuffling low, mid, and high elevation cultivars to different elevations, applying organic manure to the crop plant, etc. These possible reasons may be responsible for the upward shift in cultivation observed for Seremna and Dzongu Golsey and the downward shift for Ramsey.

Sikkim (India) lies in the mountainous region (Eastern Himalaya), and the mountainous region of the world is most vulnerable to climate change^38,39^. Studies suggest that the temperature in mountainous regions has increased rapidly compared to the Northern Hemisphere^50^ or in the lowlands^51^. In the context of India, a significant warming trend was observed during 1986–2015, with an increase in annual mean temperature by 0.15 °C, mean maximum temperature by 0.15 °C and mean minimum temperature by 0.13 °C per decade^52^. Therefore, although farmers are shuffling the cultivars in their field, climate change might augment the upward elevational shift in cultivating low-elevational cultivars like Dzongu Golsey and Seremna to mid and high elevation. A similar upward shift of 53 plant species at Mt. Gongga was observed in response to climate change^31^. They suggested that along with rising temperature^54–56^, precipitation and the functional traits of plant species such as an ability to disperse and colonize new regions effectively, also contribute to the upward migration of species along an elevational gradient^31^. Several other studies on the elevational redistribution of species in different regions have also generated a similar trend of upward shift of the plant species in response to climate change^32–37^. In the Nordic region (Northern Europe), the grass and the cereal distribution is expected to shift by up to 92.8 and 178.7 km, respectively, depending on the extent of the climate change scenario^29^ and some crop species are being introduced to new areas^30^. Such pieces of evidence of climate change have led to significant shifts in the elevational distributions of mountain species^57–59^. Hence, performance evaluation of different large cardamom cultivars in new habitats is necessary to determine their suitability and augment productivity.

The previous study suggested that elevation and plantation types contribute significantly to the variability of chlorophyll content and NDVI in large cardamom^53^. In this study, we found a significant phenotypic variability within each cultivar of the large cardamom cultivated at different elevations ranging from 975 to 2069 m. This result suggests the influence of the elevation on phenotypic traits evaluated (except for capsule length and width) in each of the five cultivars of the large cardamom. Similar results of a significant influence of elevation on biochemical constituents, nutritive value, plant height, grain yield, biological yields, and other morphological characteristics on crop plants such as rice (*Oryza sativa* L.)^60^, pea (*Pisum sativum* L.)^61^, cassava (*Manihot esculenta* Crantz)^62^, mung bean (*Vigna radiata* (L.) R.Wilczek)^42^, desho grass (*Pennisetum pedicellatum* Trin.)^63^, and the plant like pink savory (*Satureja thymbra* L.)^43^ have been reported. Phenotypic variability is highly influenced by genetic and environmental factors^64,65^. Elevation can significantly influence microclimatic conditions and environmental factors like temperature, humidity, sunlight hours, UV-B radiation, water deficits, etc^41^. For example, temperature decreases by a lapse rate of 0.52–0.65 °C with every 100 m rise in elevation^66^, while shortwave solar radiation increases with increasing elevations^67,68^. Also, various environmental factors are exposed at different elevations, responding differently to the plant’s traits^42,43^. Thus, plants alter their physiological and morphological characteristics in response to environmental conditions regulated by the elevational gradients^44,45^. Therefore, a significant phenotypic variability observed within each cultivar of the large cardamom cultivated at different elevations might have arisen due to the change in the environmental factors of precipitation, temperature, and soil characteristics along elevations^69^. However, before considering our suggestion, a proper genotype-environment interaction (GEI) study on large cardamom is necessary to determine whether this variability in traits is due to genotype or environment. A trait of a cultivar that shows consistent performance in different environments is considered stable, and cultivars with such stable traits are valuable genetic resources that could be used in large cardamom breeding programs.

Based on the Large Cardamom Guide—2015^10^, we divided the large cardamom cultivar sites into two elevational ranges—mid-elevation (975–1515 m or below 1515 m) and high elevation (above 1515 m) and compared the yield-related traits for each cultivar grown at these two elevational ranges. We found higher values for yield-related traits (number of capsules per spike, number of seeds per locule, capsule length, capsule width, fresh weight of capsule, and dry weight of capsule) in Dzongu Golsey, Sawney, and Seremna cultivated at mid-elevation sites (975–1515 m) than the same cultivars grown at high elevation sites (> 1515 m). Correlation analysis also revealed a negative relationship between elevation and the yield-related traits of these three cultivars, suggesting the negative influence of elevation on yield-related traits. A study on essential cereals like rice also revealed a similar result where rice yield, effective panicles, and grain per spike decreased with increasing elevation^60^ and in mung bean, thousand-grain weight, and biological yield reduced with increasing elevation)^42^. Although an upward shift is being observed in the cultivation of low-elevation cultivars like Seremna and Golsey, these two cultivars are more likely to perform well below 1515 m elevation based on the comparison of yield-related traits in mid and high-elevation as well as the negative correlation between elevation and yield-related traits. Likewise, the Ramsey cultivar cultivated in high elevation (> 1515 m) was found to perform better than the same cultivar in mid-elevation (975–1515 m) in terms of yield-related traits. We also found a positive correlation between elevation and its yield-related traits, suggesting that the elevation positively influences yield-related traits in the Ramsey cultivar. A similar trend was previously observed in pea plants where yield-related traits such as the number of seeds per pod, fresh seed weight plant, fresh seed yield per hectare, fresh pod yield per plant, etc., cultivated at high elevations (2124 m asl) were significantly higher than the one cultivated at low to mid-elevation (1508 m and 1645 m)^61^. The Varlangey cultivar can be grown in mid and high elevations^5,8,10,11^. In this study, it was reported from the high elevation sites (> 1515 m), and a significant moderate positive correlation was observed between elevation and the yield-related traits. Both Varlangey and Ramsey cultivars showed better performance at sites with elevation above 1515 m (high-elevation), and the positive correlation observed between elevation and yield-related traits in these two high-elevation cultivars is also indicative that these two high-elevation cultivars might further shift upward in their elevation preference, probably in response to the climate change. Although Varlangey can be grown in mid-elevation, but, it performs better in high elevation^5^ which supports our results that the Varlangey cultivar is found suitable and productive at high elevations in terms of height (tallest among the large cardamom cultivars), bearing a maximum number of leaves, maximum average values for yield-related traits like the number of capsules per spike, capsule length, capsule width, fresh capsule weight, dry capsule weight, fresh seed weight and second highest value for dry seed weight compare to other four cultivars. The highest number of sites (16 sites) in our study was occupied by Varlangey. A study in Kalimpong District in West Bengal (India) also found that most farmers prefer the Varlangey cultivar more than other cultivars in terms of productivity because it produces higher yield, matures early, and bears bigger capsule size, making it easy to harvest^70^. Our result also supports the farmer’s perception of yield in the Varlangey.

Moreover, observations of this study highlight that (1) there is a high phenotypic diversity within and among different cultivars of the large cardamom that can be exploited for developing improved cultivars through breeding programs; (2) there was a gradual upward shift in the cultivation of low elevation cultivar like Seremna and Dzongu Golsey and downward shift in the cultivation high elevation cultivar, especially Ramsey most probably due to climate change and by the new method of farming; (3) the elevation influences the yield-related traits of the different cultivars of the large cardamom; (4) Seremna, Dzongu Golsey, and Sawney cultivated in mid-elevation (below 1515 m) performed better than same cultivars found in high-elevation (above 1515 m) and Ramsey and Varlangey cultivated at an elevation above 1515 m (high-elevation) performed better than below 1515 m (mid-elevation). Therefore, our study suggests that elevation determines the productivity of the large cardamom crop and considering suitable elevational range for cultivation of different cultivars is crucial for better yield and productivity. However, our study area was limited to Sikkim Himalaya, and only the effect of elevation was considered in the analysis as an environmental factor. Therefore, more detailed studies with larger cardamom cultivation areas and the inclusion of the influence of edaphic, micro-climatic, and other environmental factors on the yield of the large cardamom along the elevational gradient are required to strengthen our observations. Nonetheless, the adaptability of large cardamom cultivars to a wide range of elevations (above and below the preferred elevations) indicates their resilience to the changing climate, which can be explored in the future to develop it as a climate-resilient crop.

## Materials and methods

### Study area

**Figure 4:**
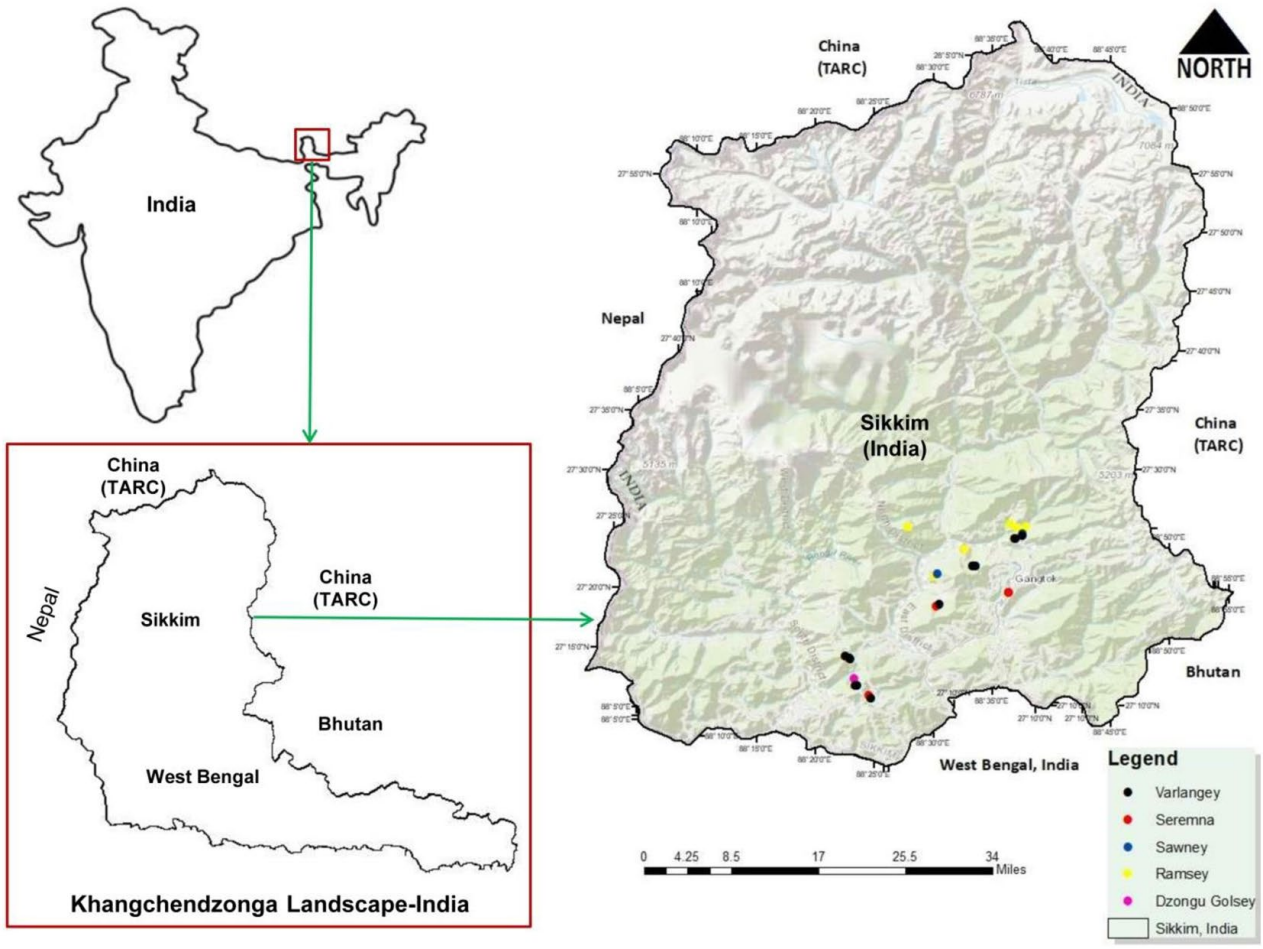
Map of study area under KL-India depicting plantation sites of five cultivars of large cardamom (Dzongy Golsey, Sawney, Seremna, Ramsey, and Varlangey) in three districts of Sikkim (East, South, and North) using ArcGIS 10.3 version.

Sikkim (27° N latitude and 88° E longitude), with a total geographical area of 7096 km^2^—is one of the states of India which falls within the Khangchendzonga Landscape (KL) region (Fig. 4) and is also a part of the Eastern Himalayas—one of the biodiversity hotspots of the world. The state is endowed with rich biodiversity, and agriculture is the primary source of livelihood for more than 64% of its population^71^. For the present study, we randomly selected 41 large cardamom cultivation sites located at three districts of Sikkim (East, North, and South) with latitude, longitude, and elevation of the overall study sites ranging between 27°14′00.2′′ N and 27°25′23.8′′ N, 88°22′32.4′′ E and 88°37′50.6′′, and 975–2069 m asl, respectively. The details of the sites, the name of the site, district, elevation, coordinates, and varieties available in each site are provided in Table S1. All the study sites (East, North, and South districts of Sikkim) experience a monsoonal climate with three distinguished seasons: summer (March–May), rainy (June–October), and winter (November to February). All the sites had an average annual rainfall of 3045.48 mm, humidity between 57.17 and 90.98%, and temperature between 11.37 and 18.08 °C based on Meteorological Station, Gangtok-Sikkim during the study period (2020 and 2021). The meteorological data of the three districts of Sikkim (North, South, and East districts of Sikkim where the study sites are located) retrieved from the Meteorological Station, Gangtok-Sikkim is given in Table 4. Sikkim was selected as the study area because large cardamom is the major cash crop in Sikkim and contributes significantly to the national large cardamom production.

**Table 4:** The meteorological data (rainfall, relative humidity, and temperature) of the three districts of Sikkim (North, South, and East) where the study sites are located) retrieved from the Meteorological Station, Gangtok-Sikkim.

District	Year	Rainfall (mm)	Relative humidity (%)	Temperature
East Sikkim	2020	2850.30	66.30–91.60	Min: 9.08 °C; Max: 15.83 °C
	2021	2794.10	54.60–90.50	Min: 10.25 °C; Max: 16.16 °C
South Sikkim	2020	3448.20	58.00–90.60	Min: 14.25 °C; Max: 21.91 °C
	2021	1959.60	44.60–88.30	Min: 15.08 °C; Max: 22.58 °C
North Sikkim	2020	3933.10	64.90–91.60	Min: 9.5 °C; Max: 15.83 °C
	2021	3287.60	54.60–93.30	Min: 10.08 °C; Max: 16.16 °C

### Data collection

Extensive field surveys were performed in 41 large cardamom cultivation sites in the three districts of Sikkim (East, South, and North Districts), out of which Dzongu Golsey was found at four sites, Sawney at five sites, Seremna at six sites, Ramsey at ten sites, and Varlangey at sixteen sites (Table S1). A total of 122 plant samples of five large cardamom cultivars, namely*,* Varlangey (48), Ramsey (30), Seremna (18), Sawney (14), and Golsey (12) (Table S2) were analyzed during the March to October months of the year 2020 and 2021. Data for twelve morphometric traits such as plant height, number of leaves per tiller, leaf length, leaf width, number of capsules per spike, capsule length, capsule width, fresh capsule weight, dry capsule weight, number of seeds per locule, fresh seed weight, and dry seed weight were recorded from the field as per the descriptor^11^. Plant height, leaf length, and leaf width were determined using measuring tape, while capsule length and capsule width were measured using a digital vernier caliper (MITUTOYO 500-197-20). To measure fresh capsule weight, 10 fresh capsules from each bush were taken randomly, and the weight was recorded using a digital weighing balance. Moreover, dry capsule weight was determined by taking the weight of the identical 10 capsules after drying in an oven. Similarly, fresh seed weight was determined by weighing 100 random fresh seeds, and dry seed weight was determined by weighing the 100 seeds after proper oven-drying in a digital weighing balance. The number of seeds per locule was recorded by taking an average of the number of seeds present in each of the four locules of the capsule. We used a manual measuring and counting method for data scoring as relevant for the traits. Data on plant height, number of leaves, leaf length, leaf width, etc., were recorded on the field without harming/destroying the plant. For measuring the length, width, and weight of capsules and seeds, we collected the capsules of the large cardamom from the field following institutional, national, and international guidelines and legislation with proper consent/permission from the concerned land owners/farmers of the study sites.

### Statistical analysis

Those mentioned above twelve morphometric data sets acquired from the large cardamom cultivation sites were observed as non-normal data sets, which were transformed to a normal distribution using arcsin transformation. The transformed data set with normal distribution was used for parametric tests like one-way analysis of variance (ANOVA), Tukey’s test, and correlation. All statistical analyses, such as descriptive statistics (minimum, maximum, average, standard error), coefficient of variation (CV), one-way ANOVA, and correlation were performed using the R program^72^.

### Supplementary Information

Supplementary Tables.

## Data Availability

Data used in the study is provided in supplementary information (Table S2).
